# Efficient querying of genomic reference databases with *gget*

**DOI:** 10.1093/bioinformatics/btac836

**Published:** 2023-01-05

**Authors:** Laura Luebbert, Lior Pachter

**Affiliations:** Division of Biology and Biological Engineering, California Institute of Technology, Pasadena, CA 91125, USA; Division of Biology and Biological Engineering, California Institute of Technology, Pasadena, CA 91125, USA; Department of Computing and Mathematical Sciences, California Institute of Technology, Pasadena, CA 91125, USA

## Abstract

**Motivation:**

A recurring challenge in interpreting genomic data is the assessment of results in the context of existing reference databases. With the increasing number of command line and Python users, there is a need for tools implementing automated, easy programmatic access to curated reference information stored in a diverse collection of large, public genomic databases.

**Results:**

*gget* is a free and open-source command line tool and Python package that enables efficient querying of genomic reference databases, such as Ensembl. *gget* consists of a collection of separate but interoperable modules, each designed to facilitate one type of database querying required for genomic data analysis in a single line of code.

**Availability and implementation:**

The manual and source code are available at https://github.com/pachterlab/gget.

**Supplementary information:**

Supplementary data are available at *Bioinformatics* online.

## 1 Introduction

The increasingly common use of genomic methods, such as single-cell RNA-seq, to provide transcriptomic characterization of cells is dependent on quick and easy access to reference information stored in large genomic databases such as Ensembl, NCBI and UniProt (Cunningham *et al.*, 2022; NCBI Resource Coordinators, 2013; UniProt Consortium, 2021). Although integrated information retrieval systems date back to the 1990s (Etzold *et al.*, 1996; Zdobnov *et al.*, 2002), a majority of researchers currently access genomic reference databases to annotate and functionally characterize putative marker genes through manual web access (Birney *et al.*, 2004; Stalker *et al.*, 2004). This process is time-consuming and potentially error-prone, as it requires manually copying and pasting data, such as gene IDs.

To facilitate and automate functional annotation for genomic data analyses, we developed *gget*: a free and open-source software package that queries information stored in several large, public databases directly from a command line or Python environment. *gget* consists of a collection of tools designed to perform the database querying required for genomic data analysis in a single line of code. In addition to providing access to genomic databases, *gget* can also leverage sequence analysis tools, such as BLAST (Altschul *et al.*, 1990, 1997), thus simplifying complex annotation workflows.

While there are other web-based application programming interface (API) data mining systems, we identified some limitations in such tools, including limits to query types and to utilizing databases in tandem. For example, while widely used and suitable for many purposes, BioMart (Durinck *et al.*, 2005; Kasprzyk *et al.*, 2004) only queries one user-defined database at a time. Moreover, large-scale genomic data analyses, such as single-cell RNA-seq data analysis, are better served by command line APIs that can fetch data directly into programming environments.

**Fig. 1.**
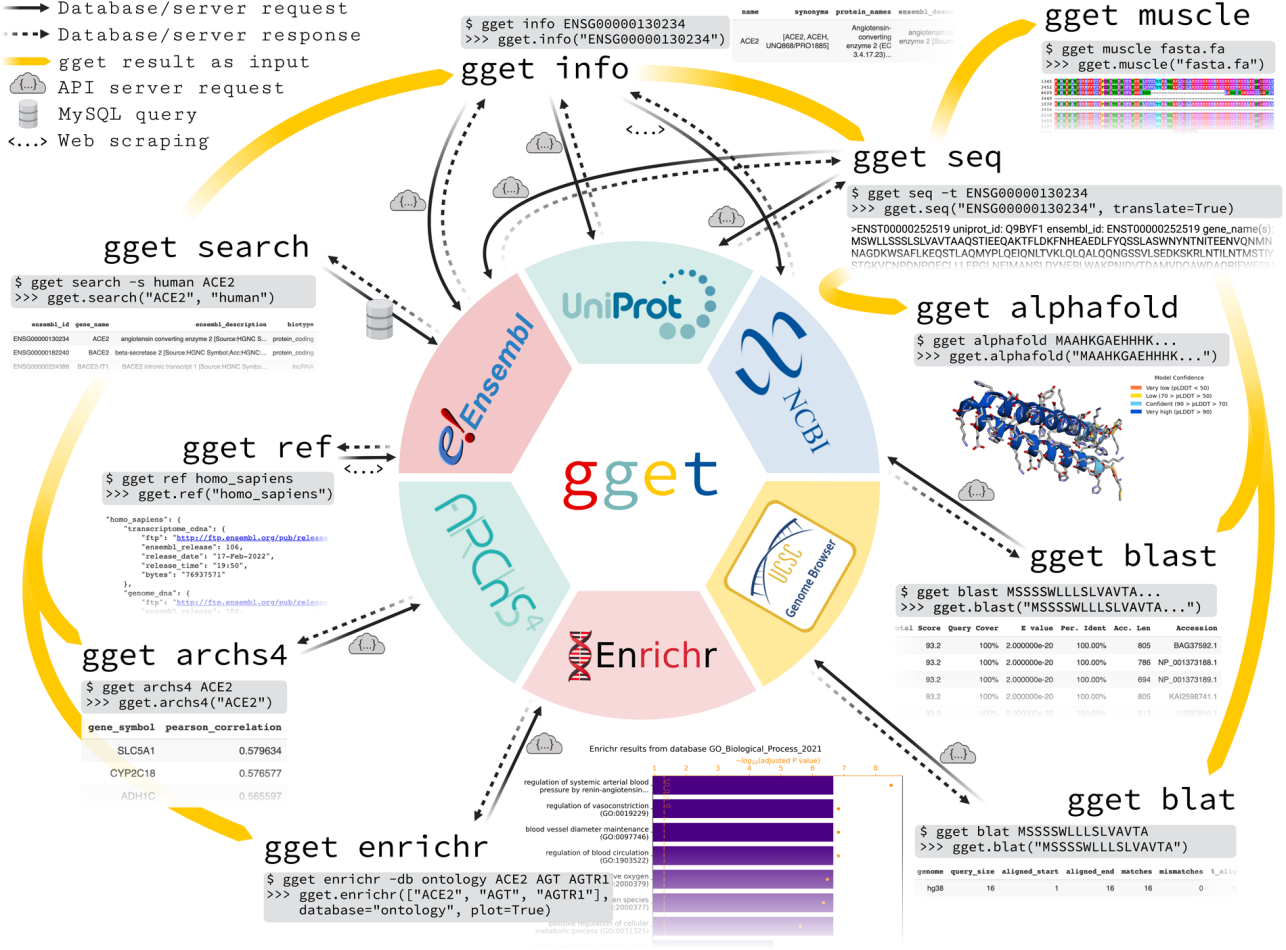
Overview of the *gget* tools and the public databases they access. One simple command line ($) example and its Python (>>>) equivalent are shown for each tool with the corresponding output.

The *gget* modules combine MySQL (Oracle Corporation, 1995), API and web data extraction queries to rapidly and reliably request comprehensive information from different databases (Fig. 1). This approach allows *gget* to perform tasks unsupported by existing tools built around standard API queries (de Ruiter, 2016). For instance, searching for genes and transcripts using free-form search terms. Each *gget* tool requires minimal arguments, provides clear output and operates from both the command line and Python environments, such as JupyterLab, maximizing ease of use and accommodating novice programmers.

## 2 Description

*gget* consists of an increasing collection of tools, currently featuring:

- *gget ref*: Fetch file transfer protocols and metadata for reference genomes or annotations from Ensembl by species.
- *gget search*: Fetch genes or transcripts from Ensembl using free-form search terms.
- *gget info*: Fetch extensive gene or transcript metadata from Ensembl, UniProt and NCBI by Ensembl ID.
- *gget seq*: Fetch nucleotide or amino acid sequences of genes or transcripts from Ensembl or UniProt by Ensembl ID.
- *gget blast*: BLAST (Altschul *et al.*, 1990, 1997) a nucleotide or amino acid sequence to any BLAST database.
- *gget blat*: Find the genomic location of a nucleotide or amino acid sequence using BLAT (James Kent, 2002).
- *gget muscle*: Align multiple nucleotide or amino acid sequences to each other using the Muscle5 algorithm (Edgar, 2021).
- *gget enrichr*: Perform an enrichment analysis on a list of genes using Enrichr (Chen *et al.*, 2013; Kuleshov *et al.*, 2016; Xie *et al.*, 2021) and an extensive collection of gene set libraries, including KEGG (Kanehisa, 2019; Kanehisa and Goto, 2000; Kanehisa *et al.*, 2021) and Gene Ontology (Ashburner *et al.*, 2000; Gene Ontology Consortium, 2021).
- *gget archs4*: Find the most correlated genes to a gene of interest or find the gene’s tissue expression atlas using ARCHS4 (Lachmann *et al.*, 2018).
- *gget pdb*: Get the structure and metadata of a protein from the RCSB Protein Data Bank (Berman *et al.*, 2000).
- *gget alphafold*: Predict the 3D structure of a protein from its amino acid sequence using a simplified version of DeepMind’s Alphafold2 (Evans *et al.*, 2022; Jumper *et al.*, 2021).

Each *gget* tool accesses data stored in one or several public databases (Fig. 1). *gget* fetches the requested data in real time, returning the latest information for each query. To ensure the accuracy of the queries, we incorporated extensive unit tests to detect changes in the API or database structures which automatically run on a bi-weekly basis. One exception to the online queries is *gget muscle*, which locally compiles the Muscle5 algorithm (Edgar, 2021) and therefore does not require an internet connection.

*gget info* combines information from Ensembl, NCBI and UniProt (Cunningham *et al.*, 2022; NCBI Resource Coordinators, 2013; UniProt Consortium, 2021) to provide the user with a comprehensive executive summary of the available information about a gene or transcript. This also enables users to assert whether data from different sources are consistent.

By accessing the NCBI server (NCBI Resource Coordinators, 2013) through HTTPS requests, *gget blast* does not require the download of a reference BLAST database, as is the case with existing BLAST tools (Buchfink *et al.*, 2021; Camacho *et al.*, 2009). The whole self-contained *gget* package is approximately 5 MB after installation.

The package dependencies were carefully chosen and kept to a minimum. *gget* depends on the HTML parser *beautifulsoup4* (Richardson, 2022), the Python MySQL-connector (Oracle, 2022) and the HTTP library *requests* (Reitz, 2022). All of these are well-established packages for server interaction in Python. *gget* has been tested on Linux/Unix, Mac OS (Darwin) and Windows.

## 3 Usage and documentation

*gget* can be installed from the command line by running ‘pip install gget’. Figure 1 depicts one use case for each *gget* tool with the corresponding output. Each *gget* tool features an extensive manual available as function documentation in a Python environment or as standard output using the help flag [-h] in the command line. The complete manual with examples can be viewed on the *gget* website, available at https://pachterlab.github.io/gget. A separate *gget examples* repository is accessible at https://github.com/pachterlab/gget_examples and includes exemplary workflows immediately executable in *Google Colaboratory* (Bisong, 2019).

## 4 Discussion

Our open-source Python and command line program *gget* enables efficient and easy programmatic access to information stored in a diverse collection of large, public genomic reference databases. *gget* works alongside existing tools that fetch user-generated sequencing data (Gálvez-Merchán *et al.*, 2022) to replace ineffective, potentially error-prone manual web access during genomic data analysis. While the *gget* modules were motivated by experience with tedious single-cell RNA-seq data analysis tasks (Supplementary Fig. S1), we anticipate their utility for a wide range of bioinformatics tasks.

## Supplementary Material

btac836_Supplementary_Data

## References

[btac836-B1] Altschul S.F. et al (1990) Basic local alignment search tool. J. Mol. Biol., 215, 403–410. 2231712 10.1016/S0022-2836(05)80360-2

[btac836-B2] Altschul S.F. et al (1997) Gapped BLAST and PSI-BLAST: a new generation of protein database search programs. Nucleic Acids Res., 25, 3389–3402. 9254694 10.1093/nar/25.17.3389 PMC146917

[btac836-B3] Ashburner M. et al (2000) Gene ontology: tool for the unification of biology. The Gene Ontology Consortium. Nat. Genet., 25, 25–29. 10802651 10.1038/75556 PMC3037419

[btac836-B4] Berman H.M. et al (2000) The Protein Data Bank. Nucleic Acids Res., 28, 235–242. 10592235 10.1093/nar/28.1.235 PMC102472

[btac836-B5] Birney E. et al (2004) An overview of Ensembl. Genome Res., 14, 925–928. 15078858 10.1101/gr.1860604 PMC479121

[btac836-B6] Bisong E. (2019) Google Colaboratory. In: *Building Machine Learning and Deep Learning Models on Google Cloud Platform: A Comprehensive Guide for Beginners*. Apress, pp. 59–64.

[btac836-B7] Buchfink B. et al (2021) Sensitive protein alignments at tree-of-life scale using DIAMOND. Nat. Methods, 18, 366–368. 33828273 10.1038/s41592-021-01101-x PMC8026399

[btac836-B8] Camacho C. et al (2009) BLAST+: architecture and applications. BMC Bioinformatics, 10, 421. 20003500 10.1186/1471-2105-10-421 PMC2803857

[btac836-B9] Chen E.Y. et al (2013) Enrichr: interactive and collaborative HTML5 gene list enrichment analysis tool. BMC Bioinformatics, 14, 128. 23586463 10.1186/1471-2105-14-128 PMC3637064

[btac836-B10] Cunningham F. et al (2022) Ensembl 2022. Nucleic Acids Res., 50, D988–D995. 34791404 10.1093/nar/gkab1049 PMC8728283

[btac836-B11] de Ruiter J. (2016) *PyBiomart 0.2.0.* https://jrderuiter.github.io/pybiomart/ (26 May 2022, date last accessed).

[btac836-B12] Durinck S. et al (2005) BioMart and Bioconductor: a powerful link between biological databases and microarray data analysis. Bioinformatics, 21, 3439–3440. 16082012 10.1093/bioinformatics/bti525

[btac836-B13] Edgar R.C. (2021) High-accuracy alignment ensembles enable unbiased assessments of sequence homology and phylogeny. 10.1101/2021.06.20.449169. PMC9664440 36379955

[btac836-B14] Etzold T. et al (1996) SRS: information retrieval system for molecular biology data banks. *Methods Enzymol*., 266, 114–128. 10.1016/s0076-6879(96)66010-8. 8743681

[btac836-B15] Evans R. et al (2022) Protein complex prediction with AlphaFold-Multimer. 10.1101/2021.10.04.463034.

[btac836-B16] Gálvez-Merchán Á. et al (2022) Metadata retrieval from genomics database with ffq. *Bioinformatics*, btac667. 10.1093/bioinformatics/btac667. PMC9883619 36610997

[btac836-B17] Gene Ontology Consortium (2021) The Gene Ontology resource: enriching a GOld mine. Nucleic Acids Res., 49, D325–D334. 33290552 10.1093/nar/gkaa1113 PMC7779012

[btac836-B18] James Kent W. (2002) BLAT—the BLAST-Like Alignment Tool. Genome Res., 12, 656–664. 11932250 10.1101/gr.229202 PMC187518

[btac836-B19] Jumper J. et al (2021) Highly accurate protein structure prediction with AlphaFold. Nature, 596, 583–589. 34265844 10.1038/s41586-021-03819-2 PMC8371605

[btac836-B20] Kanehisa M. et al (2021) KEGG: integrating viruses and cellular organisms. Nucleic Acids Res., 49, D545–D551. 33125081 10.1093/nar/gkaa970 PMC7779016

[btac836-B21] Kanehisa M. (2019) Toward understanding the origin and evolution of cellular organisms. Protein Sci., 28, 1947–1951. 31441146 10.1002/pro.3715 PMC6798127

[btac836-B22] Kanehisa M. , Goto S. (2000) KEGG: Kyoto encyclopedia of genes and genomes. Nucleic Acids Res., 28, 27–30. 10592173 10.1093/nar/28.1.27 PMC102409

[btac836-B23] Kasprzyk A. et al (2004) EnsMart: a generic system for fast and flexible access to biological data. Genome Res., 14, 160–169. 14707178 10.1101/gr.1645104 PMC314293

[btac836-B24] Kuleshov M.V. et al (2016) Enrichr: a comprehensive gene set enrichment analysis web server 2016 update. Nucleic Acids Res., 44, W90–7. 27141961 10.1093/nar/gkw377 PMC4987924

[btac836-B25] Lachmann A. et al (2018) Massive mining of publicly available RNA-seq data from human and mouse. Nat. Commun., 9, 1366. 29636450 10.1038/s41467-018-03751-6 PMC5893633

[btac836-B26] NCBI Resource Coordinators (2013) Database resources of the National Center for Biotechnology Information. Nucleic Acids Res., 41, D8–D20. 23193264 10.1093/nar/gks1189 PMC3531099

[btac836-B27] Oracle (2022) *mysql-connector-python 8.0.29*. https://dev.mysql.com/doc/connector-python/en/ (26 May 2022, date last accessed).

[btac836-B28] Oracle Corporation (1995) *MySQL*. https://www.mysql.com/ (26 May 2022, date last accessed).

[btac836-B29] Reitz K. (2022) *requests 2.27.1.* https://requests.readthedocs.io/en/latest/ (26 May 2022, date last accessed)*.*

[btac836-B30] Richardson L. (2022) *beautifulsoup4 4.11.1*. https://www.crummy.com/software/BeautifulSoup/ (26 May 2022, date last accessed).

[btac836-B31] Stalker J. et al (2004) The Ensembl Web site: mechanics of a genome browser. Genome Res., 14, 951–955. 15123591 10.1101/gr.1863004 PMC479125

[btac836-B32] UniProt Consortium (2021) UniProt: the universal protein knowledgebase in 2021. Nucleic Acids Res, 49, D480–D489. 33237286 10.1093/nar/gkaa1100 PMC7778908

[btac836-B33] Xie Z. et al (2021) Gene Set Knowledge Discovery with Enrichr. Curr Protoc, 1, e90. 33780170 10.1002/cpz1.90 PMC8152575

[btac836-B34] Zdobnov E.M. et al (2002) The EBI SRS server—recent developments. Bioinformatics, 18, 368–373. 11847095 10.1093/bioinformatics/18.2.368

