# Supplementary figures and images for: Efficient querying of genomic reference databases with gget
Source: Bioinformatics. 2023 Jan 5;39(1):btac836. doi: 10.1093/bioinformatics/btac836 (PMC9835474; doi:10.1093/bioinformatics/btac836)

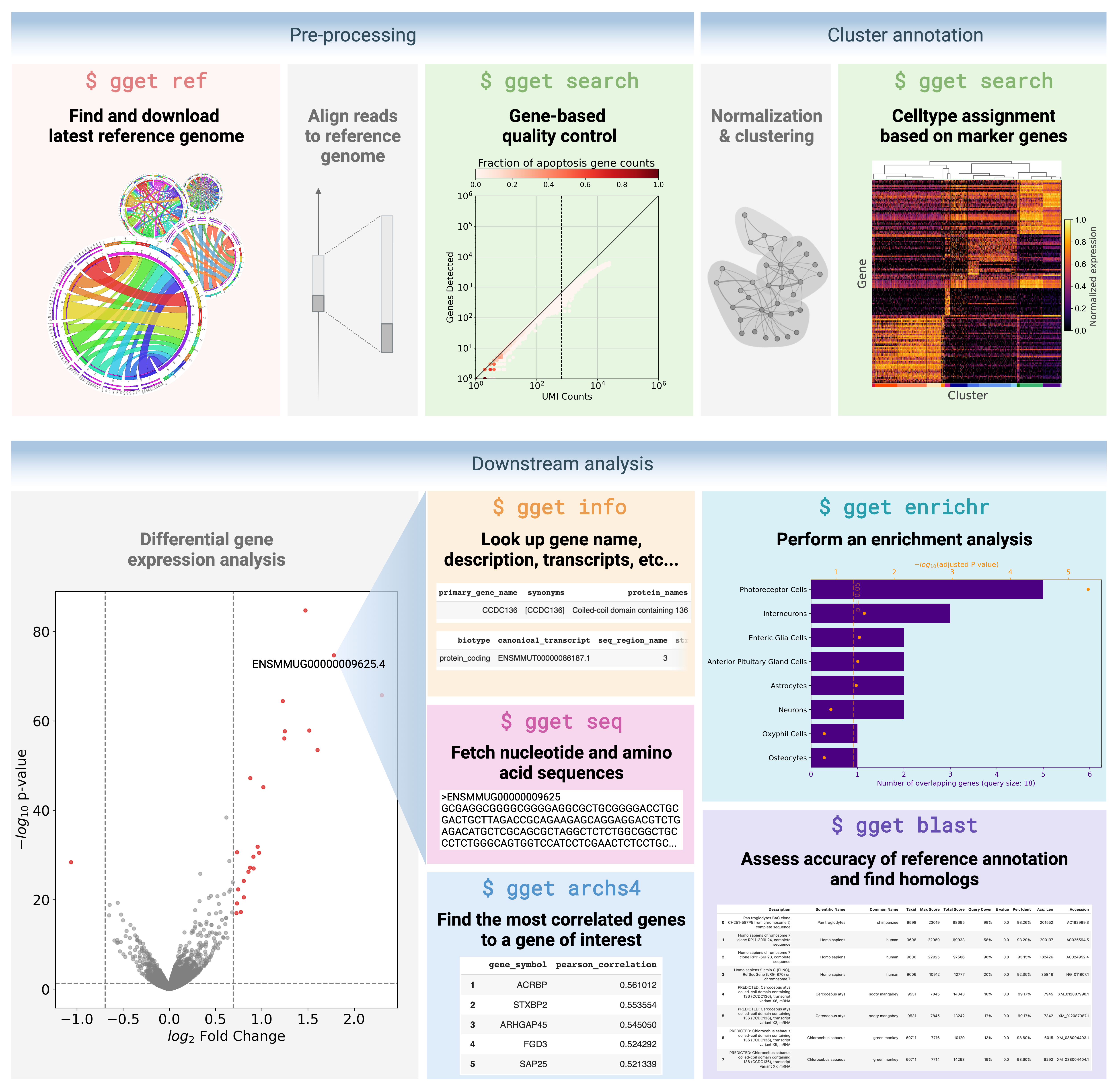

Supplement: btac836_Supplementary_Data [file btac836_supplementary_data.zip › gget_supp_fig1.png]
